# Pydna: a simulation and documentation tool for DNA assembly strategies using python

**DOI:** 10.1186/s12859-015-0544-x

**Published:** 2015-05-02

**Authors:** Filipa Pereira, Flávio Azevedo, Ângela Carvalho, Gabriela F Ribeiro, Mark W Budde, Björn Johansson

**Affiliations:** CBMA, Campus de Gualtar, University of Minho, Braga, Portugal; Division of Biology and Biological Engineering, California Institute of Technology, Pasadena, California USA

**Keywords:** Next generation cloning, Cloning simulation, Bioinformatics, Homologous recombination

## Abstract

**Background:**

Recent advances in synthetic biology have provided tools to efficiently construct complex DNA molecules which are an important part of many molecular biology and biotechnology projects. The planning of such constructs has traditionally been done manually using a DNA sequence editor which becomes error-prone as scale and complexity of the construction increase. A human-readable formal description of cloning and assembly strategies, which also allows for automatic computer simulation and verification, would therefore be a valuable tool.

**Results:**

We have developed pydna, an extensible, free and open source Python library for simulating basic molecular biology DNA unit operations such as restriction digestion, ligation, PCR, primer design, Gibson assembly and homologous recombination. A cloning strategy expressed as a pydna script provides a description that is complete, unambiguous and stable. Execution of the script automatically yields the sequence of the final molecule(s) and that of any intermediate constructs. Pydna has been designed to be understandable for biologists with limited programming skills by providing interfaces that are semantically similar to the description of molecular biology unit operations found in literature.

**Conclusions:**

Pydna simplifies both the planning and sharing of cloning strategies and is especially useful for complex or combinatorial DNA molecule construction. An important difference compared to existing tools with similar goals is the use of Python instead of a specifically constructed language, providing a simulation environment that is more flexible and extensible by the user.

**Electronic supplementary material:**

The online version of this article (doi:10.1186/s12859-015-0544-x) contains supplementary material, which is available to authorized users.

## Background

Modern biology experiments often require and depend on the construction of new DNA molecules for expression of a protein or for other cellular manipulations. While in the past, small DNA constructs incorporating few parts were common, the complexity of new constructs has grown with advancing technology. Recently, several DNA assembly protocols have been published allowing the *in-vivo* [1] or *in-vitro* [2] assembly of large DNA molecules, commonly referred to as “next generation cloning”. These protocols often describe the assembly of ten to twenty PCR generated DNA fragments into a complex construct.

The *in-silico* planning of these constructs is still often done manually using a DNA sequence editor. The planning stage usually results in an *ad hoc* natural language description of a cloning strategy. The complexity of these strategies results in risk of cloning strategy errors and omissions. The translation of the cloning strategy into a DNA sequence is dependent on human translation, which contributes to the incompletely documented or ambiguous DNA cloning strategies found in literature.

A recent example of next generation cloning protocols described the assembly of a metabolic pathway consisting of three genes permitting *S. cerevisiae* growth on the pentose sugar xylose [1]. Briefly, genes were fused with promoter and terminator sequences by fusion PCR and each promoter-gene-terminator subsequently joined into a vector by *in-vivo* recombination. Fifty-nine primers were required for the assembly, the sequences of which were given as supplementary data to the article. Our attempt to recreate the assembly *in-silico* revealed that the sequences of three primers were incomplete in such a way that they do not anneal with the designated template, possibly due to truncation of primer sequences.

Authors sometimes submit sequences which reflect the final result of the cloning strategy to a public database such as Genbank. While helpful, the strategy is still separate from the final result and intervening steps are not available for inspection or for another lab to build upon. This ambiguity and incompleteness is both unfortunate and unnecessary since the whole DNA construction process is deterministic, as there is typically only one desired way for the DNA fragments to combine and one final DNA sequence.

Properly documenting and *in-silico* simulation of constructs is crucial during the development phase of new genetic constructs. In the case of deterministic constructs that do not rely on random assembly, the theoretical final sequence is necessary for confirmation by restriction digestion which is a faster and more cost effective method to confirm the structure of DNA assemblies [3]. A solution to these problems is a strategy description that is both readable by humans and executable by a computer to simulate the individual steps of the protocol as well as the end result. Here we describe pydna, which is a software tool that was developed to provide high level computer simulation of DNA manipulation procedures and aid the design of complex constructs. Pydna contains functionality for automated primer design for homologous recombination cloning or Gibson assembly as well as DNA assembly simulation. Pydna include data types to describe double stranded DNA and the most common unit operations performed to manipulate DNA.

Pydna can aid in the design of DNA constructions and at the same time be a compact, self contained, unambiguous plan for almost any subcloning or DNA assembly experiment. Pydna allow the mixing of different kinds of assembly protocols with classical restriction endonuclease cut and paste cloning. The execution of the code verifies the accuracy and completeness of the described strategy. All intermediate results are automatically generated and can easily be inspected. Strategies described in pydna are easy to modify if necessity arises. For instance, a strategy may have to be modified due to for example a particular DNA fragment being refractory to PCR amplification. Pydna would allow simply redesigning primers and reexecute the pydna code to verify that the strategy and all downstream steps are still correct. A strategy designed by hand would require all steps downstream of the modification to be reassessed.

## Implementation

Pydna was implemented exclusively in Python and depends mainly on the Biopython [4] and NetworkX [5] packages. Source code [6] and installers [7] are available for all systems supporting Python. Distributions of pydna are built using an automatic build system [8] which also executes a large test suite (66% coverage) that is always executed before each new release, to maintain code quality. An interactive python environment with pydna installed is available on-line [9] which allow execution of small pydna examples without software installation. Static examples are also available through a IPython notebook accessible through a web browser without the need to install software [10].

Most of the pydna functionality is implemented as methods for the Dseqrecord class, which was designed to hold sequence information necessary for describing a double-stranded DNA molecule. Objects of the Dseqrecord class can also hold metadata, such as Genbank accession numbers and features tables. The Dseqrecord class supports sequence file reading, writing and downloading from Genbank. Much of the Dseqrecord functionality reflects that of the SeqRecord class of Biopython [4].

A powerful way to make use of pydna is to install the free Anaconda Scientific Python distribution [11] providing the spyder scientific python development environment [12] in combination with the IPython [13] interactive shell. Detailed installation instructions of both Anaconda and pydna can be found in Additional file 1. A compressed folder containing a comprehensive collection of examples are available in Additional file 2.

The source code of pydna contains extensive documentation for most functions and classes. These comments are automatically built into a documentation suit after each release and can be accessed online without installing pydna [14]. Questions, comments and bug-reports should be directed to the pydna mailing list [15].

## Results and discussion

### Interactive usage

This section will show examples of pydna interactive usage aiming at assembly primer design and assembly simulation. Examples are given as excerpts from an interactive session using the enhanced IPython shell. User input are preceded by the “In” prompt with a line number such as **“In** [1]**:”** while the system response is preceded by the out prompt “Out” like “**Out** [4]**:”**. The example below shows three Genbank records downloaded from Genbank and stored as Dseqrecord objects.



Pydna provides short representations of Dseqrecord objects indicating topology (linear “-” or circular “o”) and length. The Dseqrecord object YEp24PGK describes a circular 9637 bp yeast expression plasmid, while the cyc1 describes the 330 bp linear *CYC1* yeast gene. The gfp object contains the eGFP gene from the plasmid pUG35. The eGFP gene is present on the antisense strand of the vector, so it is transformed to its reverse complement using the **reverse_complement()** method.

Sequences can be cut using restriction enzymes provided by the Biopython package [4] which is automatically installed with pydna:



The linearize method cuts the plasmid at the unique BglII site in the example above. The result is a 9641 bp linear sequence. The linearized plasmid appears larger (9641 bp) than the circular (9637 bp) since the digestion produced cohesive ends. The details of the sequence can be inspected using the seq property of the object.



### Assembly primer design

Several protocols have been developed allowing the simultaneous directed assembly of a large number of DNA fragments into a final construct in one step. Homologous recombination (HR) and Gibson assembly [2] are two commonly used techniques. HR has been used to assemble large metabolic pathways and up to 25 bacterial genome DNA fragments [16]. DNA fragments to be joined are typically transformed into an organism with an efficient HR machinery such as *S. cerevisiae. In-vivo* HR between transformed DNA fragments is thought to occur mainly by the single strand annealing pathway [17]. Thirty to fifty nucleotides of homology are required for *S. cerevisiae* to efficiently join fragments, and these regions of homology should occur at or near the DNA ends of the molecules to be joined.

Gibson assembly is a protocol for joining DNA fragments *in*-*vitro* by treatment with a mixture of T5 exonuclease, DNA polymerase and Taq DNA ligase. Gibson assembly requires 20–40 bp of perfect homology between 3’ and 5’ ends for fragments to be joined. The T5 exonuclease chews back each fragment in the 5’-3’ direction so that the remaining 3’ single stranded overhangs can anneal. Gaps are filled and nicks sealed by polymerase and ligase.

The few lines of pydna code shown so far have established three linear DNA fragments, the expression vector YEp24PGK, which was linearized with BglII, and the cyc1 and gfp genes. These three fragments could be joined together to form an expression construct where the cyc1 is fused to GFP at its three prime end. A common way to accomplish this fusion is to PCR amplify the fragments with tailed primers designed to add stretches of flanking homology to each fragment. Pydna provides the pydna **assembly_primers** function in order to automatically design tailed primers for a series of DNA fragments. The pydna code below automatically designed primers for assembly by Gibson assembly or HR. Melting temperatures and the size of the desired overlaps between sequences can be controlled by optional arguments to the function. The algorithm tries to create primers with balanced melting temperature for the annealing region. The vector backbone is indicated with the keyword **vector** as primers for this sequence is not normally desired. Primers will be designed for the assembly in the given order, so order and orientation of fragments fed to this function are important.



The last three nucleotides of the cyc1 gene containing the stop codon are removed using the slice notation common for Python sequences (**[:-3]**). The p1, p2, p3 and p4 objects contain the sequences of the newly designed primers.

### PCR simulation

Pydna offers powerful PCR simulation where tailed primers and inverse PCR on a circular template are supported. Genbank features associated with the template sequence are preserved if they are fully contained in the PCR product. In the example below, we use the two primers pairs from the previous section to create two PCR products in the form of **Amplicon** objects. These objects store rich information about the PCR simulation, such as the DNA region where the primer anneals, melting temperature of each primer and also a suggested PCR program.



### Assembly simulation

**Figure 1:**
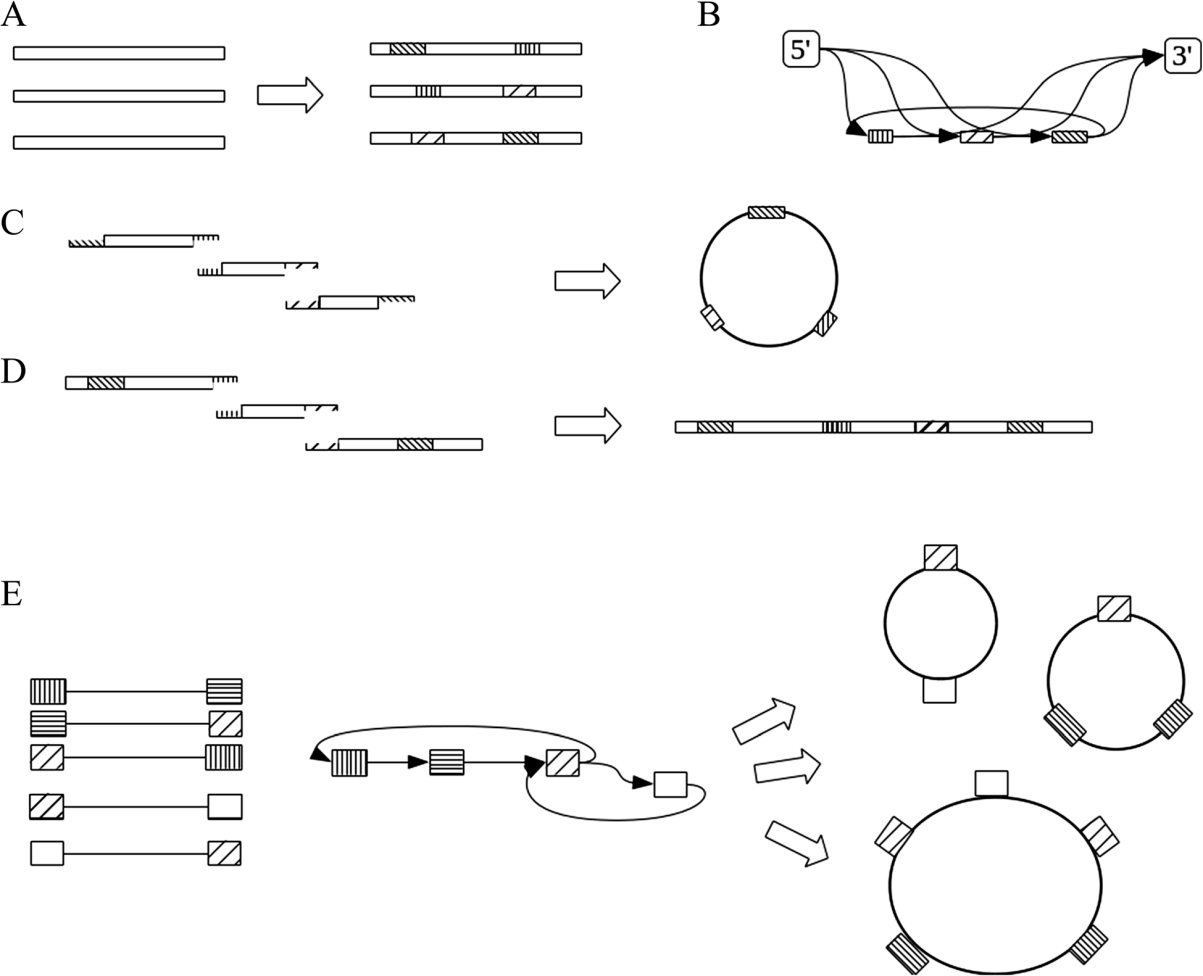
The pydna assembly process. **A)** A number of sequences (Dseqrecord objects) are fed to the algorithm and analyzed for overlapping sequences. These are added to the sequences as sequence features (striped boxes). **B)** A graph is constructed where the overlapping sequences are represented by nodes and intervening sequences are represented by edges. Two special nodes, 5′ and 3′ are added, so that the graph can be used to trace both linear and circular recombination products. **C)** A circular or linear **(D)** recombination product was found and assembled. **E)** Five sequences share homologous sequences so that the resulting graph has two circular sub graphs. All three circular graphs are returned where the largest is the combination of the two smaller sub graphs.

Pydna can automatically simulate the joining of DNA fragments by HR or Gibson assembly:



The statement above assembles the PCR products created before and stores the result in an assembly object (**asm**). The assembly algorithm is implemented in three steps. In the first step, a pairwise comparison of all sequences is performed to find shared homologous subsequences. The shared homologies are found using a pure python implementation of the fast suffix array string comparison algorithm by Kärkkäinen and Sanders [18]. Homologies are added to each sequence as metadata in the form of Genbank features (Figure 1A) which can be inspected graphically using a sequence editor.

In the second step a graph is created where overlapping sequences form nodes and sequences between overlapping sequences nodes form edges (Figure 1B). The edges of each linear fragment (5’ and 3’) are also added as nodes to the graph. The graph capabilities of Pydna are based on the widely used and thoroughly tested NetworkX graph package [5].

In the third and last step, all possible linear and circular paths are traced through the graph and the sequence of each assembly product is established. Linear graphs are all graphs between the 5’ and 3’ edges (Figure 1C and D). It is important to note that the assembly algorithm relies solely on the primary sequence of the DNA fragment with no additional constraints. The conceptual separation of the design and simulation phases that pydna can provide, improves flexibility and transparency of the simulation. All the data is stored within the **Assembly** object which can be inspected in a number of ways:



The representation of the asm object above provides a short report including the number of sequences analyzed, how many of these that share homologies. The representation also states the shortest limit considered as homology (25 bp) and whether or not internal overlaps were considered in addition to terminal overlaps. In the example above, one circular and nine linear recombined sequences were found. These are available through two properties of the **asm** object, **linear_products** and **circular_products**.



In the above statement the circular recombination product is returned as an object of the Contig class. The Contig class implements various methods and properties that allow the inspection of how the sequences were assembled. The **figure** method gives an text based figure outlining how the sequences were assembled for rapid inspection.



The figure above shows the circular molecule resulting from the assembly of the three sequences. The fragments were joined by stretches of 35 bp or 36 bp homology.

A circular molecule can be assembled from a set of existing linear sequences in several ways, if there are circular sub graphs within the main graph. Figure 1E shows a selection of five sequences with homologies represented by boxes with different patterns. The sequences share homologies in such a way that the resulting graph has two circular sub graphs. Pydna handles these by allowing one turn of each circular subgraph. In this way, the largest possible assembly product is always reported in addition to the two circular sub graphs (Figure 1E). This may not reflect the most likely outcome of an actual experiment, where products with the lowest number of participating DNA fragments are likely to be more common. However, this information is useful to catch errors in the assembly strategy on the planning stage.

### Pydna scripts

Pydna can also be used to create stand alone python scripts describing a cloning or assembly project. A pydna script can serve as a compact documentation of a cloning strategy. This section describe simulation of three different cloning strategies. The two first strategies, (YEp24PGK-XK and pGUP1), were adapted from literature and thus represent how existing cloning strategies can be formalized with pydna. The last example is an advanced assembly project of a two gene metabolic pathway conferring the ability of *S. cerevisiae* to grow on the disaccharide lactose. The lactose metabolic pathway was constructed using a combination of both cut and paste cloning and homologous recombination. Pydna scripts describing all examples are available in supplementary file 2.

### Construction of the YEp24PGK-XK vector by restriction digestion and ligation

**Figure 2:**
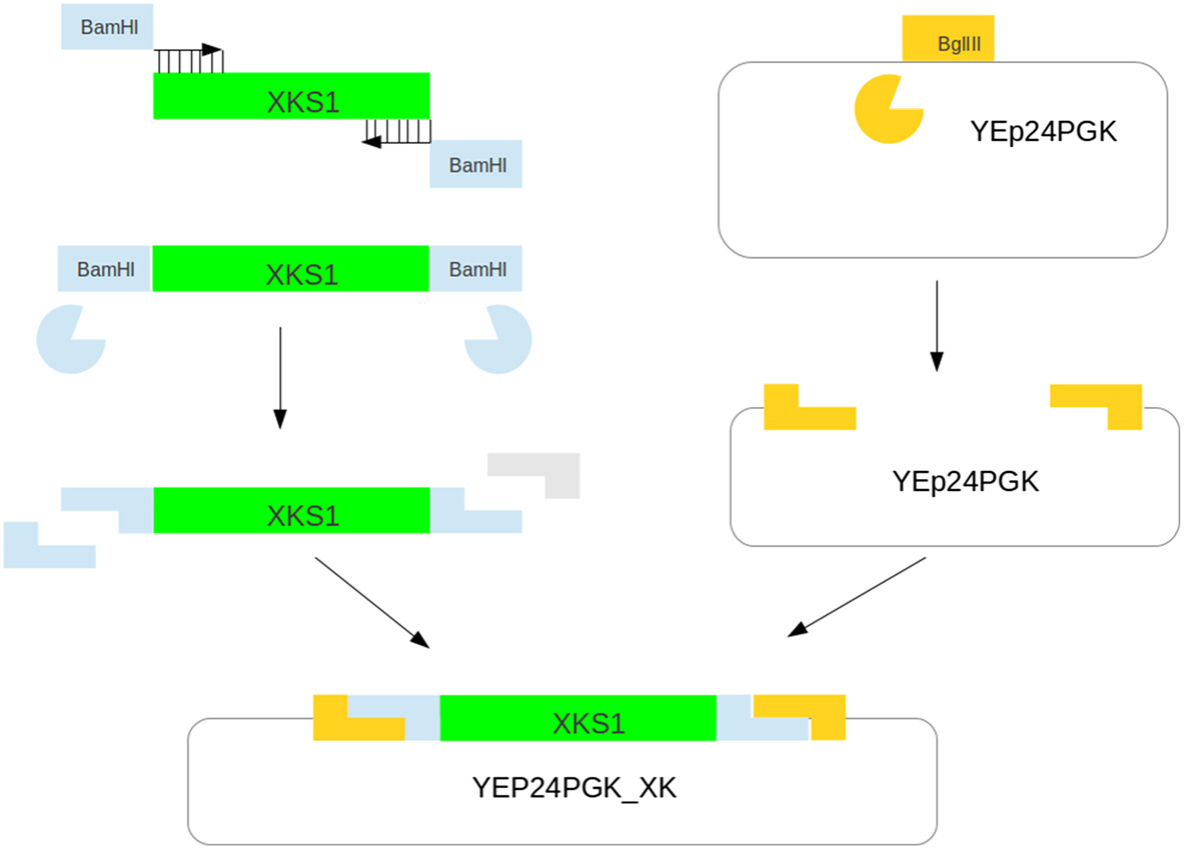
Outline of the cloning strategy described for the construction of YEp24PGK-XK. The *Saccharomyces cerevisiae* XKS1 gene was amplified by PCR from chromosomal DNA using primers 1 and 3. The PCR product was digested with BamHI and the flanking stuffer fragments removed. The vector YEp24_PGK was digested with BglII and the linear vector and the digested PCR product were ligated together using T4 DNA ligase resulting in the YEp24PGK_XK vector. The supplementary data contains a pydna script that will automatically assemble the YEp24PGK_XK vector.



The construction of the *E. coli*/*S. cerevisiae* shuttle vector YEp24PGK_XK expressing the *S. cerevisiae* xylulose kinase gene was described by Johansson and co-workers [19]. The strategy is outlined in Figure 2. Briefly, the *XKS1* gene from *S. cerevisiae* was amplified by PCR using primer1 and primer3, adding restriction sites for BamHI to the ends of the PCR product by tails added to the primers. The PCR product was digested with BamHI and ligated to the YEp24PGK plasmid (Genbank accession Z72979) that has previously been digested with BglII which cut the plasmid in one location and is compatible with BamHI. The following pydna script initiates the primer1 and primer3 sequences which were published [19], downloads the XKS1 gene sequence from Genbank, and simulates PCR. The resulting PCR product is digested with BamHI and only the middle fragment is retained. The YEp24PGK plasmid is downloaded from Genbank and digested with BglII. The digested fragments are then combined and ligated to form a circular DNA molecule. The cloning strategy can be described in twelve lines of pydna code:

The resulting circular sequence YEp24PGK_XK is 11452 bp long. The script only references sequences from Genbank, which provide a stable repository for sequences so the only information necessary to recreate the YEp24PGK_XK vector sequence is contained in the script.

### Construction of the pGUP1 vector by homologous recombination

**Figure 3:**
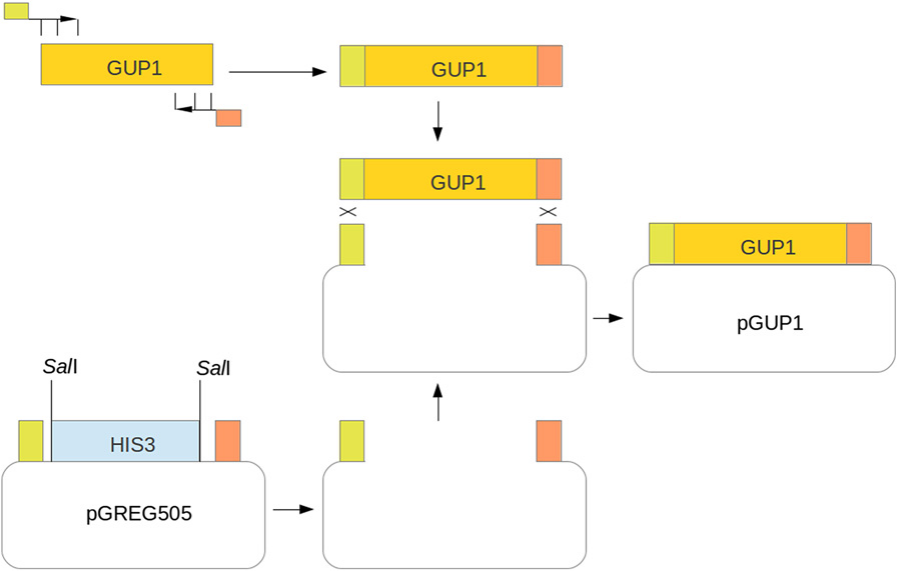
Outline of the cloning strategy described for the construction of pGUP1. The *Saccharomyces cerevisiae GUP1* gene was amplified with primers GUP1rec1sens (green) and GUP1rec2AS (red). The plasmid vector pGREG505 was digested with SalI that cuts the vector in two locations flanking the *HIS3* marker. The PCR product is joined by *in-vivo* homologous recombination to the linear vector fragment aided by short stretches of homology introduced in the PCR process.



The construction of the vector pGUP1 by homologous recombination was described by Bosson and co-workers (14). The strategy is outlined in Figure 3. The open reading frame of the *S. cerevisiae* gene *GUP1 (YGL084C)* was amplified by PCR using tailed primers GUP1rec1sens and GUP1rec2AS. The plasmid pGREG505 was digested in two locations with restriction endonuclease SalI, removing the *HIS3* marker gene. The tailed primers introduced terminal sequences with homology to the ends of the linearized vector. The two fragments were joined by homologous recombination creating the pGUP1 plasmid. The following Pydna script simulates the strategy in eleven lines of code:

The resulting circular pGUP1 plasmid has 9981 base pairs. In this example, the pGREG505 plasmid sequence is read from a local file as this sequence is not available from Genbank.

### Assembly of the lactose pathway

**Figure 4:**
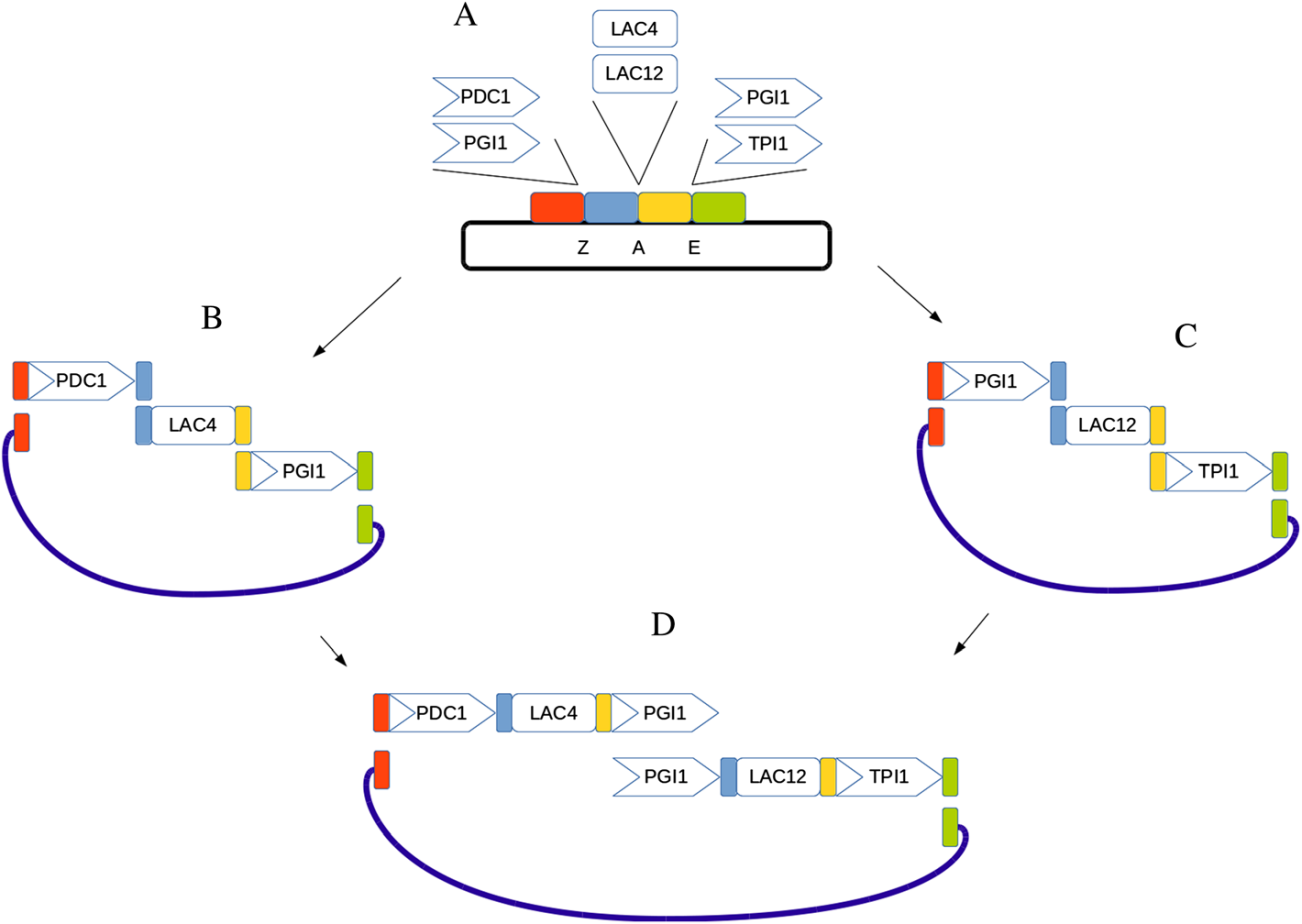
Outline of the strategy to create a lactose metabolic pathway. Six cloning vectors were constructed from five PCR products and the pYPKa vector linearized using blunt restriction enzymes ZraI, AjiI or EcoRV **(A)**. Two S. cerevisiae expression vectors were formed by homologous recombination between three PCR products and a linearized pYPKpw vector **(B and C)**. The thick blue line represents the linear pYPKpw vector. The two expression cassettes were fused by homologous recombination into a two gene expression vector **(D)**.

The advantage of a tool such as pydna is evident for the planning of larger constructs. An example of this is a heterologous lactose metabolic pathway for *S. cerevisiae* that was made from the *Kluyveromyces lactis* genes *LAC*4 and *LAC*12 encoding β-Galactosidase and a lactose permease, respectively. The genes were combined with three different promoters from the *S. cerevisiae* glycolytic genes *PDC*1, *PGI*1 and *TPI*1. All five sequences were cloned in the positive selection vector pYPKa at three different locations using blunt restriction sites specific for the ZraI, AjiI or EcoRV restriction enzymes (Figure 4A), resulting in six unique vectors (*PGI*1 promoter was cloned twice in different locations). Six PCR products were generated from the vectors allowing homologous recombination between three sequences (Figure 4B and C) and a linear *S. cerevisiae* vector (pYPKpw, thick blue line in Figure 4). Finally, linear sequences derived from the two yeast vectors were assembled into one construct (Figure 4D). This strategy has the complexity that is characteristic of multigene assemblies, that are both difficult to effectively plan and document.

A practical approach for larger projects that do not involve combinatorial assembly is to separate the construction of each physical DNA molecule in a separate pydna script. These scripts can be imported into other scripts describing other molecules as needed using the module system already present in Python, providing a way to reuse pydna code.

**Figure 5:**
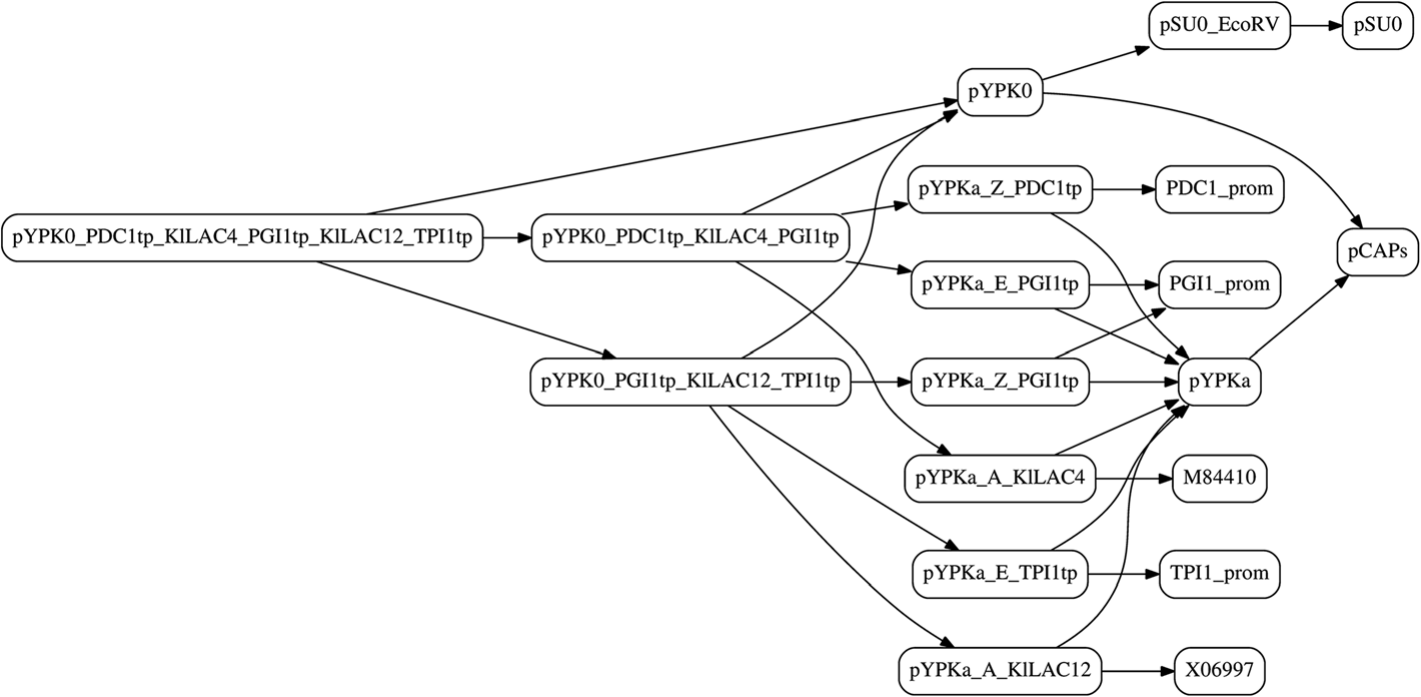
A dependency graph produced from the Lactose pathway pydna source files. Each node represents a pydna source code file with the same name and a “.py” extension. See supplementary data for further details.

The lactose pathway was described by nineteen short pydna scripts (Additional file 2) that documents the strategy starting from four Genbank sequence entries: LAC4 (M84410) [20], LAC12 (X06997) [21], pCAPs (AJ001614) [22] and pSU0 (AB215109) [23]. This example is too computationally intensive for the pydna live console and requires a local pydna installation. A cloning strategy expressed as a collection of interdependent pydna scripts can be visualized using a tool for exploring software dependencies. A graph (Figure 5) was automatically generated from the pydna scripts with no extra input. Each node in the graph represents one of the pydna scripts and the pedigree of each molecule is easily traced using the graph. Execution of the pYPK0_PDC1tp_KlLAC4_PGI1tp_KlLAC12_TPI1tp.py script describing the final pathway (Figure 4D and the left most node in Figure 5) yields the sequence of the final construct.

Another way to create an effective workflow for complex constructs is to use the IPython notebook where code text and results can be freely mixed [24]. The IPython notebook has gained popularity in the last few years as a way to develop and communicate scientific calculations with Python [25]. An example of the same lactose pathway assembly is available in supplementary file 2.

As proof of concept, the lactose pathway was created in the laboratory following the described assembly strategy. The obtained construct supported growth of *S. cerevisiae* on lactose (results not shown), which is a carbon source not naturally metabolized by this organism [26].

### Comparison with existing tools

Gibthon (https://github.com/Gibthon/Gibthon), j5 [27], and RavenCAD [28] are examples of software that were developed to solve similar problems as pydna, but with a different approach. Gibthon is free open source, while j5 and RavenCAD has some licensing restrictions although they are for now free for academic users. The j5 and RavenCAD are online tools which provide high level functionality such as optimization of cost and part reuse. More importantly, both RavenCAD and j5 are mainly meant to be used through a graphical user interface, although at least j5 can be used remotely as a command line tool through a series of Perl scripts in contrast with pydna, which was designed purely as a command line tool. This can be an advantage or disadvantage depending on the chosen workflow. A point and click graphical user interface (GUI) may have a lower initial learning threshold, while pydna requires some knowledge of Python. However, pathway assembly is inherently complex and a GUI may not be the best choice for this kind of task. Both RavenCAD and j5 rely on carefully edited data files for entering raw data, while a pydna script can be built bottom-up adding complexity gradually, iterating between coding and testing. RavenCAD leverages a specifically designed rule based language called Eugene that was designed to provide a way to create designs as scripts. While these tools are valuable in many cases, pydna will ultimately be more flexible has as it is built on top of a general purpose programming language. As mentioned, Pydna permit integration with the IPython notebook, which is a format for writing dynamic documents where code text and figures can be combined. This format has quickly gained traction especially in the scientific computation community. An example relevant for Biology is the Department of Energy Knowledgebase (http://kbase.us) offers a notebook based environment called “Narrative Interface” that leverage the flexibility of Python for different kinds of biological problems.

Any part of a pydna script can be modified to reflect changes made during implementation of the assembly in the laboratory. For example if a PCR primer that is generated automatically does not work, a replacement can be designed and the pydna script can be edited accordingly so that the strategy change is preserved in the code itself. Ultimately, the choice of tools depend on the specific requirement of each use case.

## Conclusions

The functionality provided by pydna facilitates writing compact code for describing and simulating cloning or DNA construction experiments. As the code semantically mimics molecular biology unit operations, the code is reasonably easy to read even for non-programmers. Executing the script describing a cloning strategy yields the DNA sequence of the final construct and all intermediate sequences if so required. In this way, pydna supports the same functionality as provided by some dedicated DNA sequence editors (15). Additionally, the script is a stable, verifiable and unambiguous description of a sub cloning experiment or a vector construction protocol that it simulates. This is especially true if the DNA sequences used are downloaded from Genbank, since Genbank records are guaranteed to be stable over time.

## Availability and requirements

**Project name:** pydna

**Project home page:**https://pypi.python.org/pypi/pydna

**Operating system(s):** Platform independent

**Programming language:** Python

**Other requirements:** Python 2.7

**License:** FreeBSD

**Any restrictions to use by non-academics:** None

## Additional files

Additional file 1:
**Detailed step by step instructions for the installation of the Anaconda python distribution on the Windows operating system and step by step execution of examples contained in Additional file** 2. This file is in OpenDocument Format, readable by Microsoft Word and LibreOffice writer. Additional file 2:
**This compressed file has a folder structure containing six examples of pydna usage, including the code used to produce the examples depicted in this article.**
