# Supplementary material for: Pydna: a simulation and documentation tool for DNA assembly strategies using python
Source: BMC Bioinformatics. 2015 May 2;16(1):142. doi: 10.1186/s12859-015-0544-x (PMC4472420; doi:10.1186/s12859-015-0544-x)
Supplement: Additional file 2: — This compressed file has a folder structure containing six examples of pydna usage, including the code used to produce the examples depicted in this article. [file 12859_2015_544_MOESM2_ESM.zip › supplementary data/Lactose_pathway/PCR primer design.html]

PCR primer design


# Lactose Pathway Primer Design¶

The assembly of the Lactose pathway relied on vectors and primers that were reused from other assembly projects. However, primers for for the open reading frames of the genes LAC4 and LAC12. This IPython notebook describe a workflow for primer design.

In [1]:

```
import pydna
```

Change the email address to your own. Note that NCBI require an email address of its users so that they can contact users about potential problems.

In [2]:

```
gb = pydna.Genbank("myemail@gmail.com")
```

### Primer design for the LAC4 gene¶

In [18]:

```
from IPython.display import HTML
```

In [20]:

```
HTML('<iframe src=http://www.ncbi.nlm.nih.gov/nuccore/M84410 width=800 height=350></iframe>')
```

Out[20]:

In [3]:

```
lac4 = gb.nucleotide("M84410")
```

In [4]:

```
lac4
```

Out[4]:

```
Dseqrecord(-3703)
```

The lac4 object is of the Dseqrecord class which has many useful methods. One such method is the “list\_feature” method. The size of the M84410 is 3703 bp which should of course be the same as indicated on the NCBI website.

In [5]:

```
lac4.list_features()
```

Out[5]:

```
+----------+-----------+-------+------+--------+--------------+--------------+------+
| Feature# | Direction | Start | End  | Length | id           | type         | orf? |
+----------+-----------+-------+------+--------+--------------+--------------+------+
| 0        |    -->    |   0   | 3703 |   3703 | <unknown id> | source       |  no  |
| 1        |    -->    |   8   | 3406 |   3398 | <unknown id> | gene         |  no  |
| 2        |    -->    |   8   |  12  |      4 | <unknown id> | TATA_signal  |  no  |
| 3        |    -->    |   42  | 3120 |   3078 | <unknown id> | CDS          | yes  |
| 4        |    -->    |  3400 | 3406 |      6 | <unknown id> | polyA_signal |  no  |
+----------+-----------+-------+------+--------+--------------+--------------+------+
```

The features are numbered 0-4 and the feature #3 of type “CDS” seems indeed to encode an open reading frame (ORF). The interesting sequence is extracted using the extract\_feature method and a new Dseqrecord object lac4\_orf is created.

In [6]:

```
lac4_orf=lac4.extract_feature(3)
```

In [7]:

```
lac4_orf.isorf()
```

Out[7]:

```
True
```

The pydna function print\_primer\_pair can be used to design two primers for the sequence. We add two nucleotides in front of the start codon for improved expression in Saccharomyces cerevisiae.

In [8]:

```
pydna.print_primer_pair(lac4_orf, fp_tail="aa")
```

Out[8]:

```
>pfw3078
aaATGTCTTGCCTTATTCC

>prv3078
TTATTCAAAAGCGAGATC
```

These primers above were copied and pasted into the script “pYPKa\_A\_KlLAC4.py” in the lactose\_pathway folder.

### Primer design for the LAC12 gene¶

In [21]:

```
HTML('<iframe src=http://www.ncbi.nlm.nih.gov/nuccore/X06997 width=800 height=350></iframe>')
```

Out[21]:

In [9]:

```
lac12 = gb.nucleotide("X06997")
```

In [10]:

```
lac12.list_features()
```

Out[10]:

```
+----------+-----------+-------+------+--------+--------------+---------------+------+
| Feature# | Direction | Start | End  | Length | id           | type          | orf? |
+----------+-----------+-------+------+--------+--------------+---------------+------+
| 0        |    -->    |   0   | 7127 |   7127 | <unknown id> | source        |  no  |
| 1        |    -->    |  990  | 1007 |     17 | <unknown id> | misc_feature  |  no  |
| 2        |    -->    | <1484 | 1485 |      1 | <unknown id> | precursor_RNA |  no  |
| 3        |    -->    | <1488 | 1489 |      1 | <unknown id> | precursor_RNA |  no  |
| 4        |    -->    | <1493 | 1494 |      1 | <unknown id> | precursor_RNA |  no  |
| 5        |    -->    | <1495 | 1496 |      1 | <unknown id> | precursor_RNA |  no  |
| 6        |    -->    |  1615 | 3379 |   1764 | <unknown id> | CDS           | yes  |
| 7        |    -->    | <3948 | 5217 |   1269 | <unknown id> | CDS           |  no  |
+----------+-----------+-------+------+--------+--------------+---------------+------+
```

In [11]:

```
lac12_orf = lac12.extract_feature(6)
```

In [12]:

```
lac12_orf.isorf()
```

Out[12]:

```
True
```

In [13]:

```
lac12_orf
```

Out[13]:

```
Dseqrecord(-1764)
```

In [14]:

```
pydna.print_primer_pair(lac12_orf, fp_tail="aa")
```

Out[14]:

```
>pfw1764
aaATGGCAGATCATTCGA

>prv1764
TTAAACAGATTCTGCCTC
```

The primer design was repeated for the LAC12 gene and the resulting primers were copied to the pYPKa\_A\_KlLAC12.py file.
