# Supplementary material for: Pydna: a simulation and documentation tool for DNA assembly strategies using python
Source: BMC Bioinformatics. 2015 May 2;16(1):142. doi: 10.1186/s12859-015-0544-x (PMC4472420; doi:10.1186/s12859-015-0544-x)
Supplement: Additional file 2: — This compressed file has a folder structure containing six examples of pydna usage, including the code used to produce the examples depicted in this article. [file 12859_2015_544_MOESM2_ESM.zip › supplementary data/Lactose_pathway/Assembly of the Lactose Metabolic Pathway pYPK0_PDC1_KlLAC4_PGI1_KlLAC12_TPI1.html]

Assembly of the Lactose Metabolic Pathway pYPK0\_PDC1\_KlLAC4\_PGI1\_KlLAC12\_TPI1


# Assembly of the Lactose Metabolic Pathway pYPK0\_PDC1\_KlLAC4\_PGI1\_KlLAC12\_TPI1¶

The first thing to do is importing pydna and setting an email address for using NCBI web services. If you execute this notebook, make sure to change the email address to your own. NCBI terms of service require them to have an active email address for communicating with their users.

In [1]:

```
import pydna
gb = pydna.Genbank("myemail@myemail.com")
```

Fetch the Genbank file where Kluyveromyces LAC4 gene is contained.

In [2]:

```
lac4 = gb.nucleotide("M84410")
lac4
```

Out[2]:

```
Dseqrecord(-3703)
```

The sequence we fetched is linear and 3703 bp long. We can list the features with the list\_features method.

In [3]:

```
lac4.list_features()
```

Out[3]:

```
+----------+-----------+-------+------+--------+--------------+--------------+------+
| Feature# | Direction | Start | End  | Length | id           | type         | orf? |
+----------+-----------+-------+------+--------+--------------+--------------+------+
| 0        |    -->    |   0   | 3703 |   3703 | <unknown id> | source       |  no  |
| 1        |    -->    |   8   | 3406 |   3398 | <unknown id> | gene         |  no  |
| 2        |    -->    |   8   |  12  |      4 | <unknown id> | TATA_signal  |  no  |
| 3        |    -->    |   42  | 3120 |   3078 | <unknown id> | CDS          | yes  |
| 4        |    -->    |  3400 | 3406 |      6 | <unknown id> | polyA_signal |  no  |
+----------+-----------+-------+------+--------+--------------+--------------+------+
```

The feature table shows that the feature number three is of type Coding sequence (CDS) and is an ORF (Open Reading Frame). This feature starts at position 42 and ends at 3120. We can extract this sequence by referring the the feature number in the leftmost column.

In [4]:

```
lac4_orf=lac4.extract_feature(3)
lac4_orf
```

Out[4]:

```
Dseqrecord(-3078)
```

Lets make sure this feature is a complete open reading frame in the correct orientation. The isorf method returns True if the Dseqrecord contains an open reading frame.

In [5]:

```
lac4_orf.isorf()
```

Out[5]:

```
True
```

Lets make sure the ORF encodes the protein we want. We can do this by making a protein BLAST with the translated sequence against the NCBI database.

In [6]:

```
lac4p = lac4_orf.seq.translate(cds=True)
```

The translate method returns lac4p which is a Biopython Bio.Seq object containing the sequence.

The Biopython package provide functions for remote BLAST in the NCBIWWW and NCBIXML modules.

In [7]:

```
from Bio.Blast import NCBIWWW, NCBIXML
```

In [8]:

```
result_handle = NCBIWWW.qblast("blastp", "nr", lac4p, hitlist_size=10, ncbi_gi=True)
lac4_blast_records = NCBIXML.parse(result_handle)
best_lac4_hit = lac4_blast_records.next()
best_lac4_align = best_lac4_hit.alignments[0]
```

The cell above fetches the 10 best BLAST hits from genbank. We take the first one wich seems to be a perfect match. See the Biopython cookbook for information on remote BLAST. The pydna.eq function returns True for identical sequences. The cells below shows that our translated protein sequence is the same as a Protein record in Genbank.

In [9]:

```
pydna.eq(lac4p, best_lac4_align.hsps[0].match)
```

Out[9]:

```
True
```

In [10]:

```
print best_lac4_align.title
```

```
gi|50304489|ref|XP_452194.1| hypothetical protein [Kluyveromyces lactis NRRL Y-1140] >gi|399112|sp|P00723.2|BGAL_KLULA RecName: Full=Beta-galactosidase; Short=Beta-gal; AltName: Full=Lactase [Kluyveromyces lactis NRRL Y-1140] >gi|173305|gb|AAA35265.1| beta-D-galactosidase [Kluyveromyces lactis] >gi|49641326|emb|CAH02587.1| KLLA0B14883p [Kluyveromyces lactis] >gi|608031705|gb|AHW49176.1| beta-galactosidase [Kluyveromyces lactis]
```

It appears to be the correct sequence, we can easily create a dynamic link to the web page of the protein.

In [11]:

```
print "http://www.ncbi.nlm.nih.gov/protein/"+best_lac4_align.accession
```

```
http://www.ncbi.nlm.nih.gov/protein/XP_452194
```

The strategy is to isolate this open reading frame by PCR for which we need primers. We can design PCR primers using the cloning\_primers function.

In [12]:

```
pydna.print_primer_pair(lac4_orf, fp_tail="AA")
```

Out[12]:

```
>pfw3078
AAATGTCTTGCCTTATTCC

>prv3078
TTATTCAAAAGCGAGATC
```

In [13]:

```
LAC4_CDS_f, LAC4_CDS_r = pydna.cloning_primers(lac4_orf, fp_tail="AA")
```

The forward primer is given an extra two adenines in front of the start codon as this may improve expression in Saccharomyces cerevisiae. This is dine by specifying a tail for the forward primer using the fp\_tail keyword. We can inspect the sequence of the Primers which are Biopython Bio.SeqRecord objects.

In [14]:

```
print LAC4_CDS_f.seq
```

```
AAATGTCTTGCCTTATTCC
```

In [15]:

```
print LAC4_CDS_r.seq
```

```
TTATTCAAAAGCGAGATC
```

We amplify the LAC4 gene using the new primers using the pcr function.

In [16]:

```
LAC4_prd = pydna.pcr(LAC4_CDS_f, LAC4_CDS_r, lac4)
```

The PCR product is two basepairs longer than the open reading frame as expected.

In [17]:

```
LAC4_prd
```

Out[17]:

```
Amplicon(3080)
```

We will use the pYPKa vector to clone the PCR products. This is a derivative of the pCAPs vector. The pYPKa vector is not yet available from Genbank, but it was made with site directed mutagenesis through inverse PCR. The pYPKa has a AjjI site that is not present in pCAPs. The cell below simulates the contruction of pYPKa from pCAPs.

In [18]:

```
pCAPs = gb.nucleotide("AJ001614")
primers = pydna.parse('''
>568_pCAPsAjiIR (22-mer)
GTGCcatctgtgcagacaaacg
>567_pCAPsAjiIF (23-mer)
GTcggctgcaggtcactagtgag''', ds=False)    
seq = pydna.pcr(primers, pCAPs)
seq = seq.looped()
pYPKa = seq.synced(pCAPs)
```

We will use the AjiI site to clone the LAC4 PCR product. pYPK\_A is the linear pYPK vector.

In [19]:

```
from Bio.Restriction import AjiI
pYPKa_A = pYPKa.linearize(AjiI)
```

pYPKa\_A\_LAC4 is the resulting LAC4 vector

In [20]:

```
pYPKa_A_LAC4 = (pYPKa_A + LAC4_prd).looped().synced(pYPKa)
```

We will make a similar vector for the LAC12 gene using a similar strategy. We fetch the LAC12 gene from genbank:

In [21]:

```
lac12 = gb.nucleotide("X06997")
lac12
```

Out[21]:

```
Dseqrecord(-7127)
```

This Genbank record is alslo longer than the gene we need. The ORF is marked among the features of the Genbank file.

In [22]:

```
lac12.list_features()
```

Out[22]:

```
+----------+-----------+-------+------+--------+--------------+---------------+------+
| Feature# | Direction | Start | End  | Length | id           | type          | orf? |
+----------+-----------+-------+------+--------+--------------+---------------+------+
| 0        |    -->    |   0   | 7127 |   7127 | <unknown id> | source        |  no  |
| 1        |    -->    |  990  | 1007 |     17 | <unknown id> | misc_feature  |  no  |
| 2        |    -->    | <1484 | 1485 |      1 | <unknown id> | precursor_RNA |  no  |
| 3        |    -->    | <1488 | 1489 |      1 | <unknown id> | precursor_RNA |  no  |
| 4        |    -->    | <1493 | 1494 |      1 | <unknown id> | precursor_RNA |  no  |
| 5        |    -->    | <1495 | 1496 |      1 | <unknown id> | precursor_RNA |  no  |
| 6        |    -->    |  1615 | 3379 |   1764 | <unknown id> | CDS           | yes  |
| 7        |    -->    | <3948 | 5217 |   1269 | <unknown id> | CDS           |  no  |
+----------+-----------+-------+------+--------+--------------+---------------+------+
```

The CDS number six seems to be a good candidate since it is of type CDS and it contains and ORF.

In [23]:

```
lac12_orf = lac12.extract_feature(6)
```

In [25]:

```
lac12_orf.isorf()
```

Out[25]:

```
True
```

We BLAST the lac12 protein sequence in the same way as the LAC4 gene.

In [26]:

```
lac12p = lac12_orf.seq.translate(cds=True)
```

In [27]:

```
from Bio.Blast import NCBIWWW, NCBIXML
result_handle = NCBIWWW.qblast("blastp", "nr", lac12p, hitlist_size=3, ncbi_gi=True)
blast_records = NCBIXML.parse(result_handle)
best_lac12_hit = blast_records.next()
best_lac12_align = best_lac12_hit.alignments[0]
```

In [28]:

```
print best_lac12_align.title
```

```
gi|50304487|ref|XP_452193.1| hypothetical protein [Kluyveromyces lactis NRRL Y-1140] >gi|125935|sp|P07921.1|LACP_KLULA RecName: Full=Lactose permease [Kluyveromyces lactis NRRL Y-1140] >gi|2857|emb|CAA30053.1| unnamed protein product [Kluyveromyces lactis] >gi|49641325|emb|CAH02586.1| KLLA0B14861p [Kluyveromyces lactis]
```

In [29]:

```
print "http://www.ncbi.nlm.nih.gov/protein/"+best_lac12_align.accession
```

```
http://www.ncbi.nlm.nih.gov/protein/XP_452193
```

In [30]:

```
pydna.eq(lac12p, best_lac12_align.hsps[0].match)
```

Out[30]:

```
True
```

In [31]:

```
LAC12_CDS_f, LAC12_CDS_r = pydna.cloning_primers(lac12_orf, fp_tail="AA")
```

In [32]:

```
LAC12_prd = pydna.pcr(LAC12_CDS_f,LAC12_CDS_r, lac12)
```

In [33]:

```
pYPKa_A_LAC12 = (pYPKa_A + LAC12_prd).looped().synced(pYPKa)
```

The pYPKa vector is also used to clone the promoter and terminator sequences, but other restriction sites will be used. We will use the three strong glycolytic promoters TPI1, PGI1 and PDC1 from Saccharomyces cerevisiae. We specify tails that will introduce useful restriction sites. These intergenic sequences are also fetched from genbank.

In [34]:

```
tpi = gb.nucleotide('BK006938.2 REGION: complement(556473..557055)')
tpif, tpir = pydna.cloning_primers(tpi, fp_tail="TTAAAT", rp_tail="TAATTAA")
tpi_prd = pydna.pcr(tpif, tpir, tpi)
tpi_prd
```

Out[34]:

```
Amplicon(596)
```

In [35]:

```
pgi = gb.nucleotide('BK006936.2 REGION: complement(613901..614900)')
pgif, pgir = pydna.cloning_primers(pgi, fp_tail="TTAAAT", rp_tail="TAATTAA")
pgi_prd = pydna.pcr(pgif, pgir, pgi)
pgi_prd
```

Out[35]:

```
Amplicon(1013)
```

In [36]:

```
pdc = gb.nucleotide('BK006945.2 REGION: complement(234082..235036)')
pdcf, pdcr = pydna.cloning_primers(pdc, fp_tail="TTAAATa", rp_tail="TAATTAA")
pdc_prd = pydna.pcr(pdcf, pdcr, pdc)
pdc_prd
```

Out[36]:

```
Amplicon(969)
```

In [37]:

```
from Bio.Restriction import ZraI
pYPKa_Z = pYPKa.linearize(ZraI)
```

We clone the PGI and PDC promoters in the ZraI site. This site is located a short distance before the AjiI site.

In [38]:

```
pYPKa_Z_PGI1 = (pYPKa_Z + pgi_prd).looped().synced(pYPKa)
```

In [39]:

```
pYPKa_Z_PDC1 = (pYPKa_Z + pdc_prd).looped().synced(pYPKa)
```

In [40]:

```
from Bio.Restriction import EcoRV
pYPKa_E = pYPKa.linearize(EcoRV)
```

We clone the PGI and TPI promoters in the EcoRV site. This site is located a short distance after the AjiI site.

In [41]:

```
pYPKa_E_TPI1 = (pYPKa_E + tpi_prd).looped().synced(pYPKa)
```

In [42]:

```
pYPKa_E_PGI1 = (pYPKa_E + pgi_prd).looped().synced(pYPKa)
```

We have now created six new E. coli plasmid constructs.

For the cloning of expression cassettes, we will use a derivative of pYPKa called pYPKpw that can replicate in Saccharomyces cerevisiae. This is important since in-vivo gap repair will be used. The pYPKpw is not available from Genbank, but it is based on the pSU0 and pCAPS vectors. The cell below describes the pYPKpw vector:

In [43]:

```
pSU0 = gb.nucleotide("AB215109")
primers = pydna.parse('''
>470_pSU0f-dEcoRV (50-mer)
gtttactaaaaacacatgtggatattttgactgatttttccatggagggc
>469_pSU0r-dEcoRV (50-mer)
gccctccatggaaaaatcagtcaaaatatccacatgtgtttttagtaaac''', ds=False)
pSU0_EcoRV = pydna.pcr( primers, pSU0)[50:].looped().synced("ggatccatcggaattcatattgaaaaagga")
from Bio.Restriction import BamHI, EcoRI, PvuI
psu, stuffer = pSU0_EcoRV.cut(BamHI, EcoRI)
pcaps = pCAPs.cut(PvuI).pop()
asm = pydna.Assembly2((psu, pcaps), limit=200)
pYPK0 = asm.circular_products[0]
primers = pydna.parse('''
>pYPKpwR
GCATGACGTCaccagacgctatgactcacccggacggca
>pYPKpwF
GCATGATATCttcacaggcggttttcgcacgtacccatg''', ds=False)
seq = pydna.pcr(primers, pYPK0)
seq = seq.looped()
pYPKpw = seq.synced(pYPK0)
```

The in vivo gap repair will join genes, promoters and terminators together, three and three. A set of standard primers numbered 468 to 578 will be used to produce PCR products with compatible flanking sequences.

The locations of standard primers on the pYPKa vector.

```
                             >-gene-->
            >-TP-->           \     /           >-TP-->
             \   /             \   /             \   /
              \ /               \ /               \ /
               |                 |                 |
 577>      775>| 468>       <567 | 568>       <467 |<778    <578 
 |||       ||| | |||         ||| | |||         ||| | |||     |||    
 --------------Z-----------------A-----------------E---------------------
|              r                 j                 c                     |
|              a                 i                 o                     |
|              I                 I                 R                     |
|                                                  V                     |
 --------------------- pYPKa / pYPK0 ------------------------------------
```

These primers are initiated below:

In [44]:

```
p577, p567, p468, p467, p568, p578, p775, p778 =  pydna.parse(''' 
>577_crp585-557 (29-mer)
gttctgatcctcgagcatcttaagaattc
>567_pCAPsAjiIF (23-mer)
GTcggctgcaggtcactagtgag
>468_pCAPs_release_fw (25-mer) 79.66
gtcgaggaacgccaggttgcccact
>467_pCAPs_release_re (31-mer) 
ATTTAAatcctgatgcgtttgtctgcacaga
>568_pCAPsAjiIR (22-mer)
GTGCcatctgtgcagacaaacg
>578_crp42-70 (29-mer)
gttcttgtctcattgccacattcataagt
>775_tp_ZraI_fwd (18-mer)
gcggccgctgacTTAAAT
>778_tp_Eco32I_rev (20-mer)
ggtaaatccggatTAATTAA
''', ds=False)
```

The pYPKpw is digested with EcoRV prior to gap repair.

In [45]:

```
pYPKpw_E = pYPKpw.linearize(EcoRV)
```

Three PCR products are made using the standard primers, mixed with a linear vector and assembled by gap repair.

In [46]:

```
first, middle, last = pYPKa_Z_PDC1, pYPKa_A_LAC4, pYPKa_E_PGI1

first  = pydna.pcr( p577, p567, first)

middle = pydna.pcr( p468, p467, middle)

last   = pydna.pcr( p568, p578, last)

asm = pydna.Assembly2(( pYPKpw_E, first, middle, last ))
```

Only one circular product is formed

In [47]:

```
asm
```

Out[47]:

```
Assembly:
Sequences........................: [5603] [1187] [3167] [1339]
Sequences with shared homologies.: [5603] [1187] [1339] [3167]
Homology limit (bp)..............: 25
Number of overlaps...............: 4
Nodes in graph(incl. 5' & 3')....: 6
Only terminal overlaps...........: No
Circular products................: [10824]
Linear products..................: [11068] [10965] [10874] [10861] [9828] [9766] [7744] [6698] [6649] [5606] [4469] [4304] [244] [141] [50] [37]
```

In [48]:

```
pYPK0_PDC1_KlLAC4_PGI1 = asm.circular_products[0]
```

The PCR products assembles as expected. In the figure below, the LAC4 gene is the 3167 bp fragment in the middle.

In [49]:

```
pYPK0_PDC1_KlLAC4_PGI1.small_fig()
```

Out[49]:

```
 -|5603bp_PCR_prod|124
|                  \/
|                  /\
|                  124|1187bp_PCR_prod|50
|                                      \/
|                                      /\
|                                      50|3167bp_PCR_prod|37
|                                                         \/
|                                                         /\
|                                                         37|1339bp_PCR_prod|242
|                                                                            \/
|                                                                            /\
|                                                                            242-
|                                                                               |
 -------------------------------------------------------------------------------
```

We make a similar vector for the LAC12 gene.

In [50]:

```
first, middle, last = pYPKa_Z_PGI1, pYPKa_A_LAC12, pYPKa_E_TPI1

first  = pydna.pcr( p577, p567, first)

middle = pydna.pcr( p468, p467, middle)

last   = pydna.pcr( p568, p578, last)

asm = pydna.Assembly2(( pYPKpw_E, first, middle, last ))
```

In [51]:

```
asm
```

Out[51]:

```
Assembly:
Sequences........................: [5603] [1231] [1853] [922]
Sequences with shared homologies.: [5603] [1231] [922] [1853]
Homology limit (bp)..............: 25
Number of overlaps...............: 4
Nodes in graph(incl. 5' & 3')....: 6
Only terminal overlaps...........: No
Circular products................: [9137]
Linear products..................: [9381] [9278] [9187] [9174] [8496] [8097] [7371] [6693] [6281] [3919] [3034] [2738] [244] [141] [50] [37]
```

In [52]:

```
pYPK0_PGI1_KlLAC12_TPI1 = asm.circular_products[0]
```

The 1853 bp fragment is the LAC12 gene.

In [53]:

```
pYPK0_PGI1_KlLAC12_TPI1.small_fig()
```

Out[53]:

```
 -|5603bp_PCR_prod|124
|                  \/
|                  /\
|                  124|1231bp_PCR_prod|50
|                                      \/
|                                      /\
|                                      50|1853bp_PCR_prod|37
|                                                         \/
|                                                         /\
|                                                         37|922bp_PCR_prod|242
|                                                                           \/
|                                                                           /\
|                                                                           242-
|                                                                              |
 ------------------------------------------------------------------------------
```

In [54]:

```
pYPK0_PGI1_KlLAC12_TPI1 = pYPK0_PGI1_KlLAC12_TPI1.synced(pYPKa)
```

The two separate constructs containing LAC4 and LAC12 can be joined together since the teminator for the LAC4 gene is the same sequence as the promoter for the LAC12 gene. Both promoter gene terminator cassettes can be amplified and joined through this shared sequence. The PCR primer pair below are used to amplify the promoter-gene-terminato cassette from each construct.

In [55]:

```
A,B = pYPK0_PDC1_KlLAC4_PGI1, pYPK0_PGI1_KlLAC12_TPI1

cas1  = pydna.pcr( p577, p778, A)
cas2  = pydna.pcr( p775, p578, B)
```

In [56]:

```
asm = pydna.Assembly2( (pYPKpw_E, cas1, cas2), limit=61 )
```

Three circular products are formed.

In [57]:

```
asm
```

Out[57]:

```
Assembly:
Sequences........................: [5603] [5324] [3763]
Sequences with shared homologies.: [5603] [5324] [3763]
Homology limit (bp)..............: 61
Number of overlaps...............: 4
Nodes in graph(incl. 5' & 3')....: 6
Only terminal overlaps...........: No
Circular products................: [13267] [9093] [4174]
Linear products..................: [14305] [13511] [13408] [10786] [9498] [9337] [9234] [9122] [8049] [7937] [5212] [3875] [1038] [244] [141]
```

The largest circular assembly product is the correct one (13267 bp).

In [58]:

```
pYPK0_PDC1_KlLAC4_PGI1_KlLAC12_TPI1 = asm.circular_products[0]
```

In [59]:

```
pYPK0_PDC1_KlLAC4_PGI1_KlLAC12_TPI1.small_fig()
```

Out[59]:

```
 -|5603bp_PCR_prod|124
|                  \/
|                  /\
|                  124|5324bp_PCR_prod|1013
|                                      \/
|                                      /\
|                                      1013|3763bp_PCR_prod|242
|                                                           \/
|                                                           /\
|                                                           242-
|                                                              |
 --------------------------------------------------------------
```

In [60]:

```
pYPK0_PDC1_KlLAC4_PGI1_KlLAC12_TPI1 = pYPK0_PDC1_KlLAC4_PGI1_KlLAC12_TPI1.synced(pYPKa)
```

In [61]:

```
pYPK0_PDC1_KlLAC4_PGI1_KlLAC12_TPI1
```

Out[61]:

```
Dseqrecord(o13267)
```

However the smaller products are also important as they might show up as background in the gap repair process. The next largest circular ptroduct is the one below. There is a 65 bp internal homology between the cassettes that might alternatively be formed. This has a lower probability, since the 1013 bp sequence in the previous figure has to be removed bu the DNA repair machinery for this to occur.

In [62]:

```
asm.circular_products[1].small_fig()
```

Out[62]:

```
 -|5603bp_PCR_prod|124
|                  \/
|                  /\
|                  124|5324bp_PCR_prod|65
|                                      \/
|                                      /\
|                                      65|3763bp_PCR_prod|242
|                                                         \/
|                                                         /\
|                                                         242-
|                                                            |
 ------------------------------------------------------------
```

The smallest circular recombination product is shown below. This product involves only the PCR products and not the 5603 bp vector. If the PCR products contain sequences for maintenance and selection in S. cerevisiae, this would be the dominant recombination product as only two fragments are required. In this case, this will not happen as the recombination products are selected for uracile prototophy. The URA3 gene and 2µ sequence for replication are both present on the vector.

In [63]:

```
asm.circular_products[2].small_fig()
```

Out[63]:

```
 -|5324bp_PCR_prod|1013
|                  \/
|                  /\
|                  1013|3763bp_PCR_prod|65
|                                       \/
|                                       /\
|                                       65-
|                                          |
 ------------------------------------------
```

This cloning strategy was carried out in the wet lab and the correct product 13267 bp product was indeed the predominant recombination product.

## Confirmation by restriction digestion¶

Three clones of the final vector were digested with EcoRI.

In [69]:

```
from Bio.Restriction import EcoRI
```

The lengths of fragments expected to form can be easily calculated using the list comprehension below.

In [72]:

```
[ len(x) for x in sorted( pYPK0_PDC1_KlLAC4_PGI1_KlLAC12_TPI1.cut(EcoRI), key=len ) ]
```

Out[72]:

```
[124, 883, 2234, 2937, 7109]
```

In [65]:

```
from IPython.display import Image
```

The IPython notebook can display images. The Lac4\_Lac12\_EcoRI.jpg file is a local image file of a EcoRI digestion of three clones of the final construct pYPK0\_PDC1\_KlLAC4\_PGI1\_KlLAC12\_TPI1.

Loaded from left: 1. Generuler 1kb ladder 2. empty 3. pYPK0\_PDC1\_KlLAC4\_PGI1\_KlLAC12\_TPI1 clone #1 4. pYPK0\_PDC1\_KlLAC4\_PGI1\_KlLAC12\_TPI1 clone #2 5. pYPK0\_PDC1\_KlLAC4\_PGI1\_KlLAC12\_TPI1 clone #3

The three largest bands (2234, 2937, 7109) are visible in the three rightmost lanes in the gel below.

In [66]:

```
Image('Lac4_Lac12_EcoRI.jpg')
```

Out[66]:

The molecular weight marker used is displayed below.

In [68]:

```
Image("http://www.thermoscientificbio.com/uploadedImages/Products/DNA_Electrophoresis/DNA_Ladders/sm031_fam.jpg")
```

Out[68]:
