# Supplementary figures and images for: Pydna: a simulation and documentation tool for DNA assembly strategies using python
Source: BMC Bioinformatics. 2015 May 2;16(1):142. doi: 10.1186/s12859-015-0544-x (PMC4472420; doi:10.1186/s12859-015-0544-x)

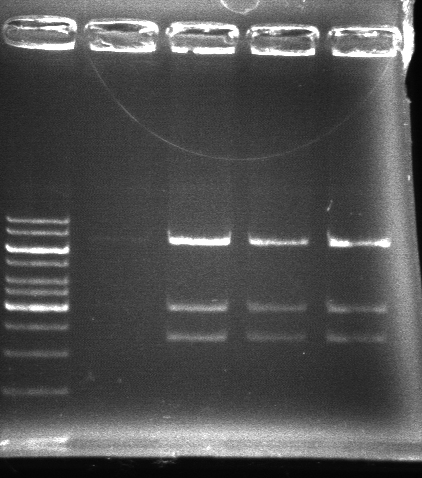

Supplement: Additional file 2: — This compressed file has a folder structure containing six examples of pydna usage, including the code used to produce the examples depicted in this article. [file 12859_2015_544_MOESM2_ESM.zip › supplementary data/Lactose_pathway/Lac4_Lac12_EcoRI.jpg]

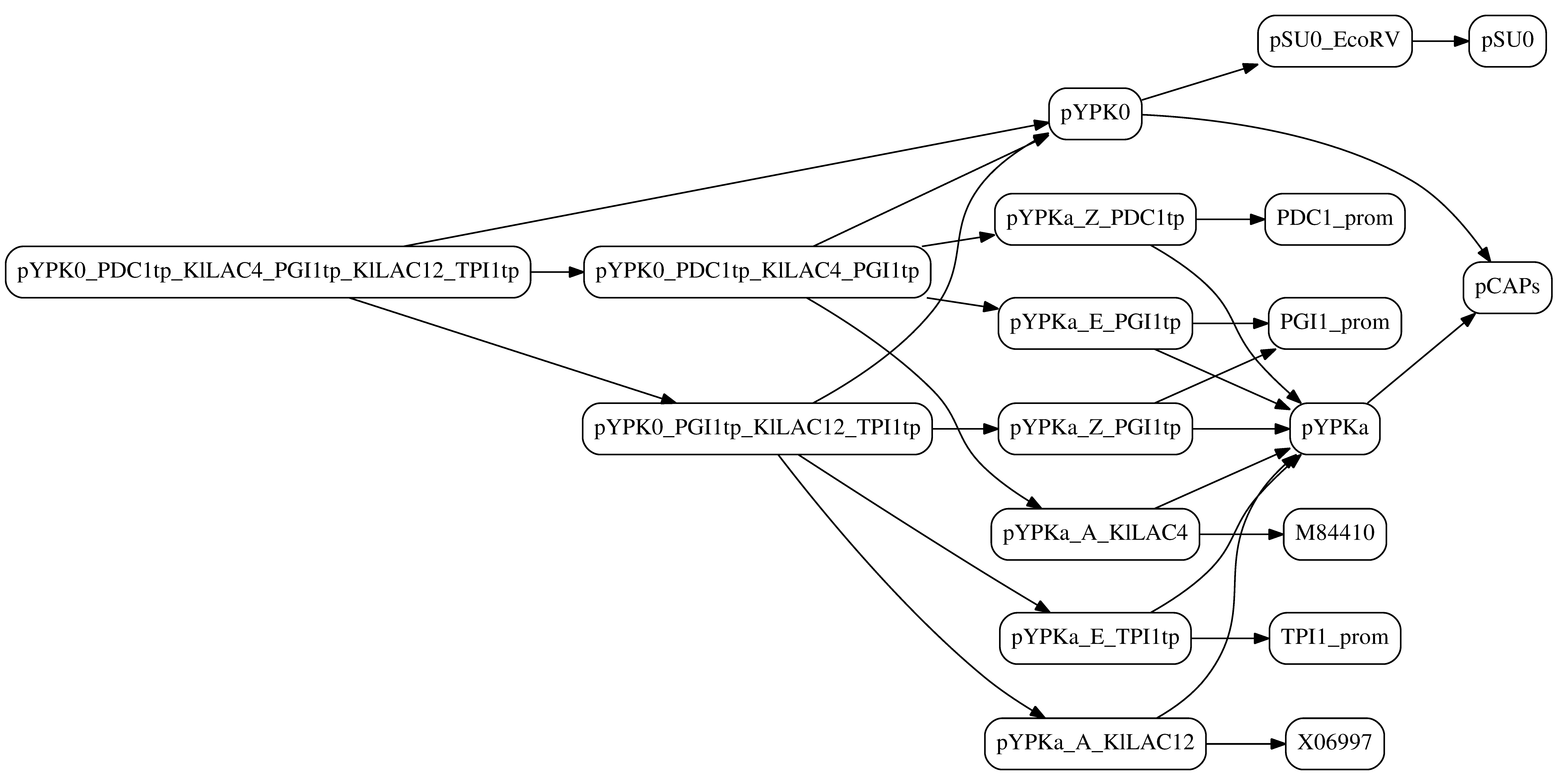

Supplement: Additional file 2: — This compressed file has a folder structure containing six examples of pydna usage, including the code used to produce the examples depicted in this article. [file 12859_2015_544_MOESM2_ESM.zip › supplementary data/Lactose_pathway/graph.png]
