# Supplementary material for: Pydna: a simulation and documentation tool for DNA assembly strategies using python
Source: BMC Bioinformatics. 2015 May 2;16(1):142. doi: 10.1186/s12859-015-0544-x (PMC4472420; doi:10.1186/s12859-015-0544-x)
Supplement: Supplementary file 90 [file 12859_2015_544_MOESM1_ESM.zip › 1990824622134218_add1.odt]

Pydna

Pydna is a software tool for the planning, simulation and documentation of metabolic pathway assemblies.

## Installation

Python 2.7 and the python packages networkx, biopython prettytable, appdirs and ipython are required to run pydna. The easiest and most efficient way to install pydna is by first installing the free Anaconda Scientific Python distribution from Continuum analytics or similar. It is a large download, but it installs cleanly in one folder in the users directory (regardless of the operating system) and is easily removable if necessary. Anaconda is available for Windows, Mac and Linux.

## Anaconda installation

Go to the website of Anaconda at [https://store.continuum.io/cshop/anaconda/](https://store.continuum.io/cshop/anaconda/) (Fig 1).

Fig 1 The Anaconda website at Continuum Analytics

Download Anaconda installation file for your operating system and follow the installation instructions. Once Anaconda is installed, find and start the Anaconda Command Prompt (see Fig 2)

Fig 2 Finding the Anaconda Command Prompt on MS Windows

The Anaconda Command Prompt starts a terminal window. Write “pip install pydna” at the command prompt followed by return (Fig 3). This command will download pydna and all dependencies and install in one go, so make sure you are connected to the internet.

Fig 3

When the installation is complete, open the Scientific PYthon Development EnviRonment (Spyder) (Fig 4).

Fig 4 Opening the Spyder (Scientific PYthon Development EnviRonment)

You should see an image like Fig 5 when Spyder is running

Fig 5 Spyder is running

The spyder window in Fig 5 show open scripts in the left pane and an open Python console in the lower right pane. IPython is a command line interface for Python that makes it easier to interact with Python. Spyder provide IPython as well as Python. Close the Python tab in the lower right corner and open an IPython console as shown in Fig 6.

Fig 6

Test that pydna was successfully installed by giving the commands “import pydna” followed by return and pydna.__version__  followed by return (Fig 7).

Fig 7

YEp24PGK_XK

We will now run some examples from the files that accompany this document. You should extract the files so that you have the folder structure for the examples shown in (Fig 8).

Fig 8

Fig 9

Go back to spyder and change the working directory to the

“Construction_of_YEp24PGK_XK_by_restriction_digestion_and_ligation” directory shown in Fig 8.

This is done by clicking the blue folder in the upper right corner of spyder (Fig 9) and navigate to where you have the folders in stored (Fig 9).

Fig 10

Use the File>open command to Open the file called   

“Construction_of_the_YEp24PGK_XK_vector.py” (Fig 11).

Fig 11

Change “[myemail@mydomain.com](mailto:myemail@mydomain.com)” to a valid email address (Fig 12).

Fig 12

Execute the script by selecting run>run or clicking the green arrow in the upper part of the spyder gui (Fig 13).

Fig 13

The output of the last print statement in the script is visible in the right lower pane of Fig 13. The resulting vector YEp24PGK_XK as well as all other variables defined in the script are available for inspection in the console (Fig 14).

Fig 14

We would perhaps want to confirm the structure of the recombinant vector by restriction digestion. The restriction enzyme HindIII cuts once in the insert and once in the YEp24PGK vector, so this enzyme could be used to distinguish the correct clones. We can test this by cutting the sequences in-silico. We import the restriction enzyme we need and confirm that the enzyme gives a distinguishable pattern of fragments for the recombinant vector (Fig 15)

Fig 15

pGUP1

This section describes the assembly of plasmid pGUP1 by homologous recombination. Execute the file “Construction_of_the_pGUP1_vector_by_homologous_recombination.py” in the folder “Construction_of_the_pGUP1_vector_by_homologous_recombination” (Fig 16)

Fig 16

We can inspect the pGUP1 variable in the console. The pGUP1 has a “small_figure” method that gives a simple ASCII representation of the assembly (Fig17).

Fig 17

The structure of the pGUP1 vector could be confirmed by restriction digestion or diagnostic PCR. One vector specific primer could be used in combination with one of the two primers used to amplify the GUP1 gene (GUP1rec1sens and GUP1rec2AS).

The primer  255_kanC 5'-TGATTTTGATGACGAGCGTAAT-3' is specific for the vector. Copy the following code into the console in order to initiate the confirmation primer (cp) (Fig 18):

cp = pydna.read('''

>255_kanC (22-mer)

TGATTTTGATGACGAGCGTAAT''', ds=False)

 

Fig 18

Copy the pydna code below to the console

pydna.pcr(cp, GUP1rec1sens, pGUP1)

The PCR product will be 2602bp long is the plasmid has the expected structure (Fig 19).

Fig 19
