# Supplementary material for: Pydna: a simulation and documentation tool for DNA assembly strategies using python
Source: BMC Bioinformatics. 2015 May 2;16(1):142. doi: 10.1186/s12859-015-0544-x (PMC4472420; doi:10.1186/s12859-015-0544-x)
Supplement: Supplementary file 91 [file 12859_2015_544_MOESM2_ESM.zip › supplementary data/Lactose_pathway/PCR primer design.odt]

PCR primer design

The assembly of the Lactose pathway relied on vectors and primers that were reused from other assembly projects. These were hard coded into the pydna scripts. 

However, there was the need to design primers for for the open reading frames of the genes LAC4 and LAC12. 

The Ipyhton notebook format provide a practical workflow for primer design (and pydna in general). An example of this is available in the file “lactose_pathway_primer_design.ipynb”. Alternatively, an IPython interactive session can be performed and saved for future reference. This document shows how to perform a pydna interactive session for primer design for the Lactose pathway.

## Genbank

If the genetic material used is avaliable in Genbank, it is best to refer directly to these records. The first step is to find the proper Genbank records for the genes of interest as both genes used are available from Genbank. 

After finding the LAC4 gene in Genbank (accession number M84410), visual inspection reveals that the Genbank file has the open reading frame marked as a feature of type CDS (Fig 1).

Fig 1 The M84410 Genbank file at NCBI

We go back to the IPython session and download the M84410 sequence using pydna (Fig 2). Note that NCBI require an email address so that they can contact users about potential problems.

Fig 2 Download of the M84410 Genbank file

The size of the M84410 is 3703 bp which should of course be the same as indicated on the NCBI website (Fig 1). The lac4 object is of the Dseqrecord class which has many useful methods. One such method is the “list_feature” method (Fig 3).

Fig 3

The features are numbered 0 - 4 and the feature of type “CDS” seems indeed to encode an open reading frame (orf). The interesting sequence is extracted using the extract_feature method by which a new Dseqrecord object lac4_orf is created.

The pydna function primer_pair (Fig 2) can be used to design two primers for the sequence.

Fig 4

The primers shown in Fig  were copied and pasted into the script “pYPKa_A_KlLAC4.py” in the lactose_pathway folder. The forward primer was modified by adding two adenines in the 5' side to potentially improve translation in S. cerevisiae. 

The primer design was repeated for the LAC12 gene and the session was saved as a html file (Fig 5). A pdf version is available as “lactose_pathway_primer_design.pdf”.

Fig 5 Saving the interactive session as html
