# Supplementary material for: Pydna: a simulation and documentation tool for DNA assembly strategies using python
Source: BMC Bioinformatics. 2015 May 2;16(1):142. doi: 10.1186/s12859-015-0544-x (PMC4472420; doi:10.1186/s12859-015-0544-x)
Supplement: Supplementary file 91 [file 12859_2015_544_MOESM2_ESM.zip › supplementary data/Lactose_pathway/How to make a dependency graph using snakefood.odt]

Dependency graph

The dependency graph software “snakefood” was used to generate a dependency graph for the pathway (Fig 1). Snakefood can be installed at the command line by typing:

pip install sfood

Snake food is a command line application that was given the following command to produce the graph:

sfood pYPK0_PDC1tp_KlLAC4_PGI1tp_KlLAC12_TPI1tp.py --internal --follow | sfood-graph --remove-extensions > graph.dot

Please refer to the snakefood [web site](http://furius.ca/snakefood/) for further details. A file called “graph.dot” is generated from the command above. This is a text file containing a graph in the GraphViz format. Graphviz is open source graph visualization software.

dot -Tps graph.dot -o graph.ps

The file graph.ps is a postscript image file that can be turned into other formats using normal image tools.

Snakefood: [http://furius.ca/snakefood](http://furius.ca/snakefood), [https://pypi.python.org/pypi/snakefood](https://pypi.python.org/pypi/snakefood) 

GraphViz: [www.graphviz.org](http://www.graphviz.org/) 

Fig 1
