# Supplementary material for: Pydna: a simulation and documentation tool for DNA assembly strategies using python
Source: BMC Bioinformatics. 2015 May 2;16(1):142. doi: 10.1186/s12859-015-0544-x (PMC4472420; doi:10.1186/s12859-015-0544-x)
Supplement: Supplementary file 91 [file 12859_2015_544_MOESM2_ESM.zip › supplementary data/Lactose_pathway/READ THIS FIRST.odt]

Files contained in this folder

This folder should contain the eleven files and folders indicated above that are all related to the Lactose pathway example. The folder “genbank_files” contain sequence files in genbank format covering all DNA fragments and vectors involved in the construction. These files were generated from the collection of pydna scripts contained in the “pydna_scripts” folder. The pydna scripts folder should contain sixteen different scripts.

These two approaches represent pydna usage with a different choice of workflow, a choice that might be dependent on scale of the project and other factors.

The “PCR primer design.odt” contains a description on how primers were designed using an interactive IPython session. “PCR primer design.ipynb” contain an IPython notebook containing the same information. There is a html copy of this file with the same name but with the .html extension for viewing.

The “Assembly of the Lactose Metabolic Pathway pYPK0_PDC1_KlLAC4_PGI1_KlLAC12_TPI1.ipynb” file contain a IPython notebook covering the same construction as the files in the “pydna_scripts” folder. The difference is that the construction is contained in a single file and comments are added to form a interactive document. There is a html copy of this file with the same name but with the .html extension for viewing.
